# Comparative Assessment of Soil Organic Carbon Stocks in Sal (*Shorea robusta*) and Mixed Hardwood Forests of the Nepal Terai

**DOI:** 10.1002/pei3.70127

**Published:** 2026-02-22

**Authors:** Ranjan Aryal, Gandhiv Kafle

**Affiliations:** ^1^ Faculty of Forestry Agriculture and Forestry University Makawanpur Hetauda Nepal

**Keywords:** bulk density, forest carbon sequestration, forest management, hardwood, terai Nepal, tropical dry forests

## Abstract

Forest soils are a critical component of the global carbon cycle, yet the impact of forest composition on soil organic carbon (SOC) stocks in tropical regions remains poorly quantified. Understanding the differences between mono‐dominant and mixed‐species forests is essential for climate change mitigation strategies. This study provides a comparative assessment of SOC stocks in two major forest types of the Nepal Terai: Sal ( *Shorea robusta* ) mono‐dominant forests and Terai Mixed Hardwood (TMH) forests. Soil samples were collected from three depth intervals (0–10, 10–20, and 20–30 cm) in five replicate plots for each forest type in Kapilvastu District. Bulk density was measured using the core method, and SOC was determined via the Walkley‐Black wet oxidation technique. SOC stocks were calculated and differences between forest types were analyzed using analysis of variance (ANOVA). The mean SOC stock in the top 30 cm was significantly higher (*p* < 0.05) in TMH forests (18.25 ± 1.2 t ha^−1^) compared to *S. robusta* forests (15.35 ± 0.9 t ha^−1^). Bulk density was significantly lower in TMH soils, while SOC concentration decreased significantly with depth in both forest types. The topsoil (0–10 cm) layer contained the largest proportion of the total SOC stock. The findings demonstrate that mixed hardwood forests in the terai Nepal store significantly greater amounts of soil carbon than *S. robusta* ‐dominated forests. This suggests that forest composition is a key determinant of carbon sequestration potential. Conservation and promotion of mixed‐species forests should be prioritized in forest management and carbon incentive programs, such as REDD+, to enhance terrestrial carbon sinks and mitigate climate change.

## 1 Introduction

Soil organic carbon (SOC) constitutes a pivotal component of the global carbon cycle, representing the largest terrestrial carbon pool with an estimated stock of 1500 to 2400 GtC in the top 1 m of soil—a quantity that significantly exceeds the combined carbon pools of the atmosphere (around 830 GtC) and terrestrial vegetation (450–650 GtC) (IPCC 2013; Scharlemann et al. 2014; Batjes 1996). Forests play a crucial role in this context, acting as major sinks for atmospheric carbon by sequestering 861 GtC globally, with biomass accounting for 363 GtC (42%) and soil storing roughly 383 GtC (44%) (Pan et al. 2011; IPCC 2013). The importance of SOC extends beyond carbon storage; it serves as a key indicator of soil health, influencing nutrient cycling, water retention, and overall ecosystem productivity (Lal 2004). With increasing concerns about climate change, understanding the dynamics of SOC stocks in different forest ecosystems has become imperative for developing effective climate change mitigation strategies.

The capacity of forests to store carbon is not uniform which varies significantly with forest type, species composition, and management practices (ICIMOD 2016). Tropical forests, in particular, are critical in the global carbon equation due to their high productivity and rapid carbon turnover rates (Gibbs et al. 2007). Recent research has highlighted that mixed‐species forests often exhibit different carbon sequestration patterns compared to mono‐dominant stands, influenced by factors such as litter quality, root architecture, and microbial community composition (Lal 2005; Prescott 2010). These differences can significantly affect both the quantity and stability of carbon stored in forest soils, making forest composition a key consideration in carbon management strategies.

The Terai region of Nepal, a tropical belt of alluvial plains, hosts critical forest ecosystems including the highly significant Sal ( *Shorea robusta* C.F. Gaertn.) forests and the more diverse Terai Mixed Hardwood (TMH) forests (Stainton 1972). *S. robusta* is a dominant, economically vital species forming monodominant stands, while TMH comprises a mix of species such as *Terminalia alata* and *Anogeissus latifolia* (DFRS 2015). These forests are not only essential for biodiversity and local livelihoods but also represent a significant national carbon stock, with Nepalese forests storing an estimated 176.95 t C/ha (DFRS 2015). The management of these forest resources has implications for both local ecosystem services and global climate change mitigation efforts.

Although numerous studies have quantified carbon stocks in Nepal, research has often focused on above‐ground biomass or national‐scale aggregates. However, recent regional assessments have begun to map the specific soil organic carbon (SOC) potential of the Terai plains. The Forest Research and Training Center (FRTC 2024) reported that the total carbon in the soil component of Terai forests is approximately 47.38 t ha^−1^, contributing significantly to the region's total carbon stock of 177.33 t ha^−1^. In the central Terai, research in the Kankali community forest of Chitwan showed that community‐managed Sal forests could store significant carbon, though stocks vary based on management intensity (Bhattarai et al. 2012).

Furthermore, landscape‐level studies in the Churia and Terai regions have identified that land‐use systems and forest types are primary determinants of SOC variability (Aulestia 2019). This trend is echoed in the global/regional scenario, specifically in India's tropical dry forests, where research demonstrates that mono‐dominant *Shorea robusta* stands exhibit different carbon sequestration patterns compared to mixed‐species hardwood stands like *Tectona grandis* (Bohre et al. 2012). Despite this wealth of regional data, a critical knowledge gap remains: there is a lack of high‐resolution, depth‐wise (0–30 cm) comparisons between mono‐dominant Sal and diverse TMH forests within the Kapilvastu district. Understanding these specific dynamics is essential to move from general national estimates to site‐specific carbon management required for REDD+ programs.

Globally, soil organic carbon (SOC) assessments employ diverse approaches ranging from large‐scale remote sensing and geostatistical modeling to localized field‐based inventories and spectroscopic techniques (Mulder et al. 2011; Viscarra Rossel et al. 2006). While predictive modeling offers broad landscape‐level estimates, empirical field‐based inventories involving core sampling and laboratory analysis (e.g., dry combustion or wet oxidation) remain the essential “gold standard” for validating regional carbon stocks in heterogeneous tropical ecosystems (Nelson and Sommers 1996). In the regional context of the South Asian Terai, the Forest Research and Training Center (FRTC 2024) recently established a regional SOC baseline of 47.38 t ha^−1^, highlighting the significant terrestrial sink potential of these forests. Research in neighboring Indian tropical deciduous forests indicates that mono‐dominant *Shorea robusta* (Sal) stands exhibit distinct carbon sequestration patterns compared to mixed hardwood stands, largely driven by variations in litter quality and root architecture (Bohre et al. 2012). Similarly, studies in the eastern Nepal Terai emphasize that species composition and management intensity are primary determinants of soil carbon stability (Gautam and Mandal 2016). Despite these regional insights, a critical knowledge gap remains regarding high‐resolution, depth‐wise (0–30 cm) comparisons between mono‐dominant Sal and diverse Terai Mixed Hardwood (TMH) forests in the central Terai. This study addresses this gap by utilizing a field‐based analytical approach, employing stratified random sampling and the Walkley‐Black wet oxidation method to provide the site‐specific ground‐truth data required for Tier 3 reporting under Nepal's REDD+ framework.

This study aims to fill this critical knowledge gap by conducting a comparative assessment of SOC stocks in *S. robusta* and TMH forests within the Kapilvastu District of Nepal. The specific objectives are: (1) to quantify the SOC stock at different soil depths (0–10, 10–20, and 20–30 cm) in both forest types, and (2) to statistically compare these stocks to determine the influence of forest composition on carbon sequestration potential. The primary hypothesis of this study is that Terai Mixed Hardwood (TMH) forests will exhibit significantly higher soil organic carbon stocks compared to mono‐dominant *Shorea robusta* forests due to niche complementarity and higher litter diversity. Conversely, the null hypothesis is that there is no significant difference in SOC stocks between the two forest types under similar physiographic conditions. By testing these hypotheses, this research expects to provide evidence‐based insights into which forest structure functions as a more effective terrestrial carbon sink, thereby guiding sustainable forest management and enhancing the accuracy of regional carbon accounting in the context of Nepal's REDD+ framework.

## 2 Methodology

### 2.1 Study Area

**FIGURE 1:**
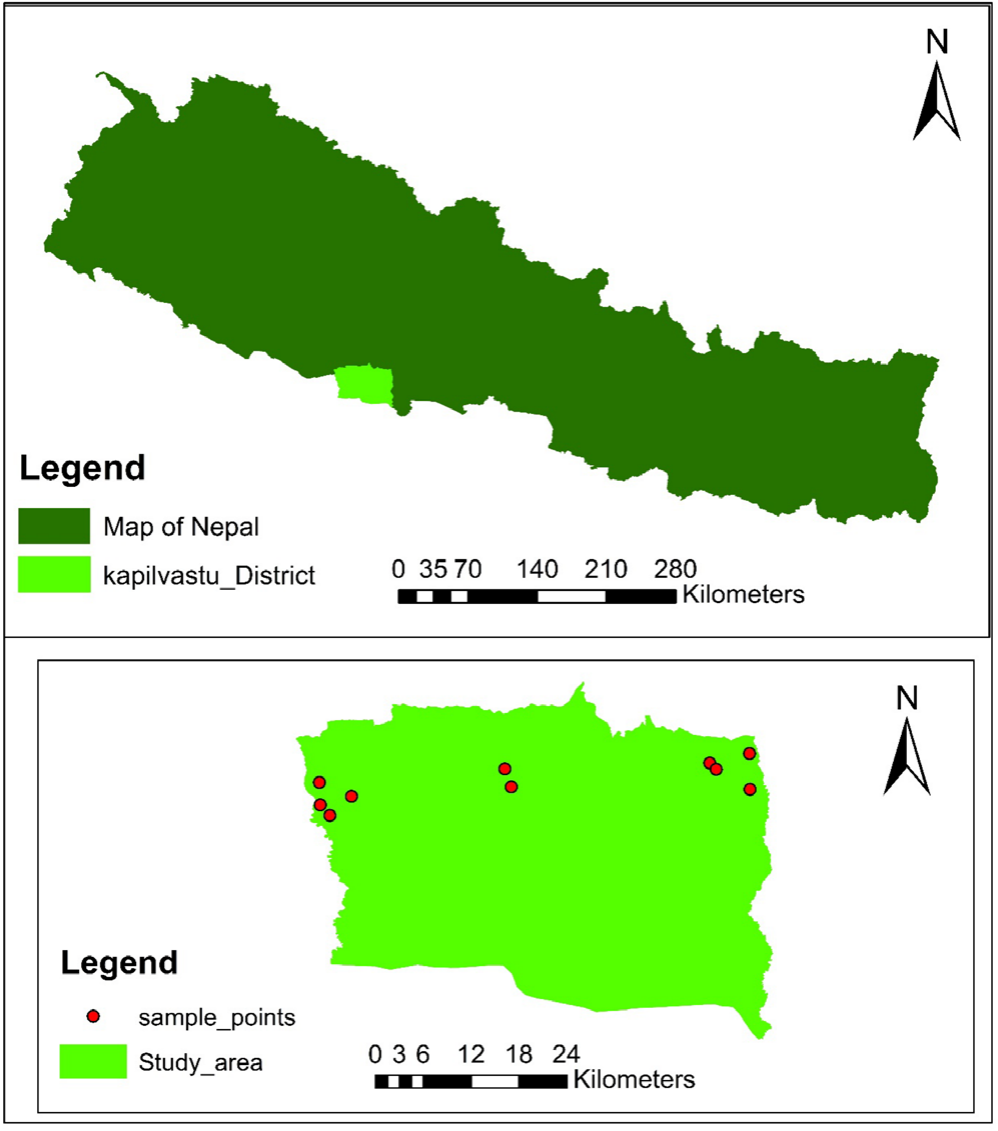
Map of study area with sampling points.

This study was conducted in the Kapilvastu District of Lumbini Province, Nepal (Figure 1). The district lies within the central Terai ecological zone, a fertile belt of alluvial plains stretching along the southern foothills of the Himalayas. Geographically, the district extends from 27°25′ to 27°84′ N latitude and 82°75′ to 83°14′ E longitude, encompassing an area of 1738 km^2^ (DFRS 2015). The topography is predominantly flat, with an elevation gradient ranging from approximately 93 m above sea level (m a.s.l.) in the southern plains to 1491 m a.s.l. in the northern Chure (Siwalik) hills.

The climate of the region is characterized as subtropical monsoon, with three distinct seasons: a hot, dry summer (March–June), a warm, wet monsoon (July–September), and a cool, dry winter (October–February). Mean annual precipitation is approximately 1200 mm, the majority of which is received during the monsoon season. Mean annual temperature is 24°C, with monthly averages ranging from 15°C in January to 32°C in May.

The dominant soil types across the study area are Fluvisols and Cambisols, developed on recent, generally deep, well‐drained, and loamy‐textured alluvial deposits (LRMP 1986; Karki et al. 2021).

The study focused on two major forest types within the district:

1.
*S. robusta* Forest: These are predominantly mono‐specific stands of *S. robusta* ., a deciduous species that dominates the landscape. The associated understory typically includes grasses such as *Imperata cylindrica and Saccharum spontaneum, along with* scattered shrubs including *Justicia adhatoda* .
2. Terai Mixed Hardwood (TMH) Forest: These forests are characterized by a greater diversity of tree species, including *Terminalia alata* Heyne ex Roth, *Anogeissus latifolia* (Roxb. ex DC.) Wall. ex Guill. & Perr., *Mallotus philippensis* (Lam.) Müll. Arg., and *Lagerstroemia parviflora* Roxb. *Shorea robusta co‐exists with these species but does not dominate the canopy*. The structure is more complex, with a multi‐layered canopy.

Forest cover in Kapilvastu District is approximately 63,438 ha, representing a significant portion of the landscape and comprising a mix of these two forest types, often managed as community forests (DFRS 2015).

### 2.2 Data Collection

#### 2.2.1 Site and Plot Selection

A comparative study design was employed to assess differences in SOC stocks between two forest types: *S. robusta* and TMH. The study area was stratified based on forest type using recent forest management plans and satellite imagery. Within each stratum ( *S. robusta* and TMH), five sample plots were randomly selected, yielding a total of 10 plots (*n* = 5 per forest type). Each circular plot had a radius of 20 m (area = 0.126 ha). Plots were selected to minimize confounding environmental variables—such as slope aspect, soil texture, and microclimatic variations—that could independently influence carbon dynamics. Efforts were made to ensure plots within each forest type were situated on similar topography (flat to gently sloping), soil types, and elevation to isolate the effect of forest composition on SOC stocks. The sampling design and plot configuration followed the National Forest Assessment standards for the Terai region, utilizing a stratified random sampling approach with 20 m radius circular plots (DFRS 2014).

#### 2.2.2 Soil Sampling Protocol

Soil sampling was conducted between January and March 2021 due to the dry season not affecting soil moisture heavily by rainfall. Within each main plot, a 2 m × 2 m sub‐plot was established 1 m outside the northern edge of the main plot to avoid soil disturbance from vegetation sampling. From the center of this sub‐plot, a single soil pit was excavated to a depth of 30 cm using a spade and sharp‐edged pickaxe for initial penetration, followed by a trowel to ensure precise vertical walls for depth measurement.

Soil samples were collected by depth increment: 0–10, 10–20, and 20–30 cm. For each depth increment, undisturbed soil cores were extracted using a stainless‐steel core sampler of known volume (100 cm^3^; AMS Inc., USA) to determine bulk density, following the core method described by Blake and Hartge (1986) and McKenzie et al. (2004). This depth‐incremental approach aligns with standard protocols for forest soil carbon inventories (Pearson et al. 2007). The sampler consisted of a stainless‐steel cylinder (5.0‐cm diameter × 5.1‐cm height) driven into the soil using a slide hammer to minimize compaction and maintain the natural soil structure.

Additionally, a composite soil sample (~500 g) for each depth was created by mixing sub‐samples taken from the four cardinal directions (North, East, South, West) within the pit wall. This ensured a representative sample of the plot's soil heterogeneity.

All samples were placed in labeled, airtight polyethylene bags. Undisturbed cores were carefully handled to maintain their structure. Samples were transported to the soil laboratory for analysis in the Forest Research and Training Center (FRTC), Kathmandu, Nepal.

### 2.3 Laboratory Analysis and Calculations

#### 2.3.1 Bulk Density (BD)

Bulk density was determined using the core method (Blake and Hartge 1986). The undisturbed soil cores were oven‐dried at 105°C for 24 h until a constant weight was achieved. Bulk density (g cm^−3^) was calculated as:

$$\mathrm{BD}=\left(\text{Oven}-\mathrm{dry}\;\text{weight of soil},g\right)/\left(\text{Volume of the core},{\mathrm{cm}}^{3}\right)$$

#### 2.3.2 Soil Organic Carbon (SOC) Concentration

The SOC concentration was determined using the wet oxidation method of Walkley and Black (1934). This method involves the oxidation of organic matter by potassium dichromate (K_2_Cr_2_O_7_) in the presence of sulfuric acid, followed by titration of the excess dichromate with 0.5 N ferrous ammonium sulfate (FAS). The percentage of SOC was calculated using the formula:

$$\%C=\left[3.951*\;\left(1-T/S\right)\right]/g$$

where *g* = weight of the soil sample (g); *T* = volume of ferrous ammonium sulfate (FAS) used in titrating the sample (mL); *S* = volume of ferrous ammonium sulfate (FAS) used in the blank titration (mL); 3.951 is a constant based on the oxidation efficiency and molecular weight of carbon.

A correction factor of 1.33 was applied to account for the presumed 77% oxidation efficiency of the Walkley‐Black method.

#### 2.3.3 Soil Organic Carbon Stock Calculation

The SOC stock (in megagrams per hectare, Mg ha^−1^, equivalent to t ha^−1^) for each depth layer was calculated using the following equation (Pearson et al. 2007):

SOCstock=BD×D×%C×100

where BD = bulk density (g cm^−3^); *D* = thickness of the soil layer (cm); %C = Soil organic carbon concentration (percent); 100 is a factor for unit conversion (to Mg ha^−1^).

The total SOC stock for the 0–30 cm profile was obtained by summing the stocks of the three individual layers.

### 2.4 Statistical Analysis

All statistical analyses were performed using R software (version 4.3.1; R Core Team 2023). Data were first screened for outliers and tested for the assumptions of normality using the shapiro.test function from the stats package. Homogeneity of variances was assessed using the leveneTest function from the car package (Fox and Weisberg 2019). To compare the mean SOC stocks and soil properties between the Sal and TMH forest types, an independent samples *t*‐test was performed using the t.test function. For depth‐wise comparisons within each forest type, a one‐way Analysis of Variance (ANOVA) was conducted via the aov function, followed by Tukey's Honest Significant Difference (HSD) post hoc test using the TukeyHSD function. All statistical significance was evaluated at a 95% confidence level (*p* < 0.05).

## 3 Results

### 3.1 Bulk Density of Forest Soils in *S. robusta* and Terai Mized Hardwood (TMH) Forests

The bulk density in *S. robusta* forest soils showed a distinct profile with depth. The highest mean BD was recorded in the topsoil (0–10 cm: 1.50 ± 0.02 g cm^−3^). The value decreased significantly in the 10–20 cm layer (1.48 ± 0.03 g cm^−3^) before increasing again in the deepest layer (20–30 cm: 1.49 ± 0.03 g cm^−3^). The overall mean BD for *S. robusta* forest across the 0–30 cm profile was 1.49 ± 0.03 g cm^−3^ (Table 1).

**TABLE 1:** Bulk density (g cm^−3^) under *S. robusta* and TMH forests at different soil depths.

Forest type	Depth (cm)	Bulk density (g cm^−3^) (mean ± SD)	Statistical significance, *p*
*S. robusta*	0–10	1.50 ± 0.02	aA, 0.041
*S. robusta*	10–20	1.48 ± 0.03	bA, 0.038
*S. robusta*	20–30	1.49 ± 0.03	abA, 0.420
TMH	0–10	1.46 ± 0.03	bB, 0.000
TMH	10–20	1.46 ± 0.03	bB, 0.999
TMH	20–30	1.50 ± 0.03	aA, 0.024

*Note:* Different lowercase letters within a column indicate significant differences between depths for a given forest type; different uppercase letters indicate significant differences between forest types at a specific depth (Tukey's HSD test, *p* < 0.05).

In contrast, TMH forests exhibited a bulk density profile. The lowest BD was found in the topsoil (0–10 cm: 1.46 ± 0.03 g cm^−3^) and it increased steadily with depth, reaching its maximum in the 20–30 cm layer (1.50 ± 0.03 g cm^−3^). The BD in the 10–20 cm layer was 1.46 ± 0.03 g cm^−3^. The overall mean BD for TMH was 1.47 ± 0.04 g cm^−3^ (Table 1).

Statistical analysis (two‐way ANOVA) confirmed that the mean bulk density was significantly higher in *S. robusta* forests compared to TMH forests (*p* < 0.05). This difference was particularly pronounced in the top 20 cm of soil. At the 0–10 cm depth, BD in *S. robusta* forest was significantly higher than in TMH forest (*p* < 0.01). Similarly, at 10–20 cm, *S. robusta* forest BD remained significantly greater (*p* < 0.05). However, at the 20–30 cm depth, there was no significant difference in BD between the two forest types (*p* > 0.05) (Table 1).

### 3.2 Soil Organic Carbon Concentration (%)of Forest Soils in *S. robusta* and Terai Mized Hardwood (TMH) Forests

Soil organic carbon concentration in *S. robusta* forests decreased markedly with increasing soil depth. The highest SOC% was found in the topmost layer (0–10 cm: 0.46% ± 0.05%). This value declined significantly to 0.32% ± 0.04% in the 10–20 cm layer and further to 0.25% ± 0.03% in the 20–30 cm layer. The mean SOC concentration for the entire profile was 0.34% ± 0.01% (Table 2).

**TABLE 2:** Soil organic carbon concentration (%) under *S. robusta* and TMH forests at different soil depths.

Forest type	Depth (cm)	SOC concentration (%) (mean ± SD)	Statistical significance, *p*
*S. robusta*	0–10	0.46 ± 0.05	aB, 0.000
*S. robusta*	10–20	0.32 ± 0.04	bA, 0.002
*S. robusta*	20–30	0.25 ± 0.03	bA, 0.046
TMH	0–10	0.69 ± 0.08	aA, 0.000
TMH	10–20	0.33 ± 0.04	bA, 0.001
TMH	20–30	0.23 ± 0.03	bA, 0.032

*Note:* Different lowercase letters within a column indicate significant differences between depths for a given forest type; different uppercase letters indicate significant differences between forest types at a specific depth (Tukey's HSD test, *p* < 0.05).

TMH forests also exhibited a decreasing trend in SOC% with depth, but with a significantly higher concentration in the topsoil. The SOC% in the 0–10 cm layer was 0.69% ± 0.08%. This value dropped sharply to 0.33% ± 0.04% at 10–20 cm and to 0.23% ± 0.03% at 20–30 cm. The profile mean SOC% for TMH was 0.42% ± 0.01% (Table 2).

The soil organic carbon concentration was significantly influenced by forest type (*p* < 0.01). The mean SOC% across the 0–30 cm profile was significantly higher in TMH forests than in *S. robusta* forests. This difference was overwhelmingly driven by the topsoil (0–10 cm), where the SOC% in TMH was approximately 50% higher than in *S. robusta* forest (*p* < 0.001). At deeper layers (10–20 and 20–30 cm), there were no statistically significant differences in SOC% between the two forest types (*p* > 0.05) (Table 2).

### 3.3 Soil Organic Carbon Stock (t/Ha) of Forest Soils in *S. robusta* and Terai Mized Hardwood (TMH) Forests

The SOC stock in *S. robusta* forests followed the same decreasing pattern with depth as the SOC concentration. The largest stock was contained in the 0–10 cm layer (6.92 ± 0.8 t ha^−1^), followed by the 10–20 cm layer (4.75 ± 0.6 t ha^−1^), and the 20–30 cm layer (3.68 ± 0.5 t ha^−1^). The total SOC stock for the 0–30 cm profile was 15.35 ± 1.2 t ha^−1^ (Table 3).

**TABLE 3:** Soil organic carbon stock (Mg ha^−1^) under *S. robusta* and Terai Mixed Hardwood (TMH) forests at different soil depths.

Forest type	Depth (cm)	SOC stock (Mg ha^−1^) (mean ± SD)	Statistical significance, *p*
*S. robusta*	0–10	6.92 ± 0.80	aB, 0.000
*S. robusta*	10–20	4.75 ± 0.60	bA, 0.003
*S. robusta*	20–30	3.68 ± 0.50	bA, 0.048
TMH	0–10	10.03 ± 1.20	aA, 0.000
TMH	10–20	4.82 ± 0.60	bA, 0.035
TMH	20–30	3.41 ± 0.40	bA, 0.000

*Note:* Different lowercase letters within a column indicate significant differences between depths for a given forest type; different uppercase letters indicate significant differences between forest types at a specific depth (Tukey's HSD test, *p* < 0.05).

In TMH forests, the SOC stock was also highest in the topsoil. The 0–10 cm layer stored 10.03 ± 1.2 t ha^−1^, which was substantially larger than the stocks in the deeper layers (10–20 cm: 4.82 ± 0.6 t ha^−1^; 20–30 cm: 3.41 ± 0.4 t ha^−1^). The total SOC stock accumulated in the top 30 cm was 18.25 ± 1.5 t ha^−1^ (Table 3).

The total soil organic carbon stock was significantly higher in TMH forests compared to *S. robusta* forests (*p* < 0.01). This significant difference was entirely attributable to the topsoil (0–10 cm), where the SOC stock in TMH was over 3 t ha^−1^ greater than in *S. robusta* forest (*p* < 0.001). The SOC stocks in the 10–20 cm and 20–30 cm layers were not significantly different between the two forest types (*p* > 0.05). Overall, TMH forests stored approximately 19% more soil organic carbon in the top 30 cm than Sal forests (Table 3, Table 4).

**TABLE 4:** Summary of total soil organic carbon stocks and profile averages.

Forest type	Total SOC stock (0–30 cm) (Mg ha^−1^) (mean ± SD)	Average SOC concentration (%) (mean ± SD)	Average bulk density (g cm^−3^) (mean ± SD)
*S. robusta*	15.35 ± 1.20	0.34 ± 0.01	1.49 ± 0.03
TMH	18.25 ± 1.50^a^	0.42 ± 0.01^a^	1.47 ± 0.04^a^
*p*	0.010	0.001	0.045

^a^ Significant difference from *S. robusta* forest at *p* < 0.05 (*t*‐test).

## 4 Discussion

This study provides clear evidence that forest composition significantly influences soil organic carbon dynamics in the Terai region of Nepal. The significantly higher SOC stock found in TMH forests (18.25 tha^−1^) compared to *S. robusta* forests (15.35 tha^−1^) underscores the importance of species diversity for soil carbon sequestration.

Our findings align with regional studies conducted in similar tropical landscapes. For instance, Bhattarai et al. (2012) observed that community‐managed forests in Chitwan exhibit varying carbon potentials based on species mix and management. Furthermore, research in India's tropical dry forests by Bohre et al. (2012) highlights that mixed‐species stands often facilitate better carbon stabilization than *Shorea robusta* monocultures due to more complex litter decomposition pathways.

The lower bulk density observed in TMH soils suggests improved soil structure and porosity. This can be attributed to a more diverse and abundant root system from multiple tree species, which enhances soil aggregation and reduces compaction (Jandl et al. 2007). In contrast, the monodominant root architecture of *S. robusta* may lead to a more homogeneous and potentially compacted soil environment. Lower BD facilitates better water infiltration, aeration, and root penetration, creating a positive feedback loop for organic matter incorporation and decomposition.

The superior SOC storage in TMH forests likely results from several interconnected factors. First, mixed‐species forests generally produce a greater quantity and diversity of litter (leaf, root, and woody debris). Different litter qualities with varying chemical compositions (e.g., C:N ratios, lignin content) can lead to more complex decomposition pathways, potentially favoring the formation of stable organic matter over simple mineralization (Prescott 2010). Second, the higher plant diversity in TMH supports a more diverse soil microbial and faunal community, which can enhance the efficiency of organic matter turnover and stabilization within soil aggregates (Lange et al. 2015). Third, it is plausible that the TMH stands in our study area experience slightly less anthropogenic pressure (e.g., litter removal, grazing) than the more heavily utilized *S. robusta* forests, which are valued for timber and fodder (Gautam and Devoe 2006). This reduced disturbance allows for greater accumulation of organic matter.

Our findings align with studies from other tropical systems that report higher carbon stocks in mixed forests compared to monocultures. However, our values (15.35–18.25 t ha^−1^) are consistent with other studies in the Terai plains (Gautam and Mandal 2016), where high temperatures and moisture during the monsoon lead to rapid decomposition compared to the cooler high‐altitude forests included in the national average (DFRS 2015).

The discrepancy between our findings (15.35–18.25 t ha^−1^) and the FRTC (2024) Terai average (47.38 t ha^−1^) can be primarily attributed to sampling depth. Over 45% of the total SOC in our study was concentrated in the top 10 cm, confirming the vulnerability and high activity of the surface layer. This surface‐heavy distribution is consistent with Paudel et al. (2016), who noted that tropical Sal forests in Nepal accumulate most of their organic matter in the upper soil horizons. The lower bulk density in TMH soils observed here (1.47 g cm^−3^) compared to Sal (1.49 g cm^−3^) suggests that mixed stands promote better soil structure, which in turn facilitates higher organic matter incorporation.

Any activity that exposes the topsoil, such as deforestation or intensive grazing, could lead to rapid oxidation and loss of this labile carbon pool to the atmosphere (Lal 2005). Therefore, conservation efforts must prioritize the protection of the soil surface.

## 5 Limitations and Implications

A limitation of this study is the relatively small sample size (*n* = 5 per forest type) within one district. While statistically significant differences were detected, future research across a wider geographical area with more replicates would strengthen these conclusions. Additionally, measuring litterfall, root biomass, and soil microbial properties would help elucidate the precise mechanisms driving the observed differences.

From a management perspective, our results suggest that promoting mixed‐species forests, rather than *S. robusta* monocultures, could be a more effective strategy for enhancing soil carbon sequestration in the Terai. This is highly relevant for Nepal's climate change mitigation policies, including REDD+. Carbon incentive programs should recognize the value of forest diversity and soil carbon pools, encouraging management practices that conserve and enhance TMH stands for their superior ecosystem service provision.

## 6 Conclusion

This study provides clear empirical evidence that forest composition is a critical determinant of soil organic carbon dynamics in the Terai region of Nepal. The comparative analysis between Sal ( *Shorea robusta* ) and Terai Mixed Hardwood (TMH) forests yielded two principal findings with significant implications.

First, the significantly higher bulk density observed in *S. robusta* forests, particularly in the topsoil, indicates a more compacted soil environment compared to the structurally superior soils of TMH forests. This suggests that the diverse root systems and potentially higher biological activity in mixed‐species stands contribute to improved soil porosity and structure. Second, and most importantly, TMH forests demonstrated a significantly greater capacity for soil carbon sequestration, storing 18.25 t C ha^−1^ in the top 30 cm compared to 15.35 t C ha^−1^ in *S. robusta* forests—a difference of approximately 19%. This enhanced carbon storage in TMH is overwhelmingly concentrated in the topsoil (0–10 cm), where carbon stocks were over 3 t C ha^−1^ greater than in *S. robusta* forests. This pattern underscores that the primary mechanism for increased carbon sequestration in mixed forests is linked to surface processes, namely the quantity and quality of litter input and the subsequent formation of soil organic matter.

The convergence of these findings—lower bulk density and higher carbon stocks in TMH—strongly suggests that mixed hardwood forests create a more favorable environment for soil carbon accumulation and stabilization. These findings provide a baseline for calculating ‘Soil Carbon Sequestration Credits’ under Nepal's REDD+ framework, suggesting that maintaining TMH diversity can increase soil‐based carbon offsets by approximately 19% compared to *S. robusta* ‐dominant stands. Policy instruments must evolve to recognize the superior ecosystem service value of diverse forests, incentivizing practices that protect soil carbon pools. Future research should focus on elucidating the mechanistic drivers, including litter decomposition rates, root biomass dynamics, and soil microbial community structure, to further optimize management for carbon sequestration.

## Funding

We are grateful to the Forest Research and Training Center (FRTC), Kathmandu, Nepal for providing a paid internship to the first author for the research.

## Conflicts of Interest

The authors declare no conflicts of interest.

## Data Availability

The data that supports the findings of this study are openly available in figshare at http://doi.org/10.6084/m9.figshare.31216117.
